# Environmentally mediated interactions predict community assembly and invasion success in a gut microbiota synthetic community

**DOI:** 10.1128/msystems.00113-26

**Published:** 2026-04-10

**Authors:** P. T. van Leeuwen, P. Gadaleta, S. Brul, J. Seppen, M. T. Wortel

**Affiliations:** 1 Molecular Biology and Microbial Food Safety, Swammerdam Institute for Life Sciences, University of Amsterdam 215691, Amsterdam, the Netherlands; 2 Tytgat Institute for Gastrointestinal and Liver Disease, Amsterdam UMC 522567, Amsterdam, the Netherlands; Boston College, Chestnut Hill, Massachusetts, USA

**Keywords:** human gut microbiome, invasion stability, synthetic community, generalized Lotka-Volterra, community predictions

## Abstract

The gut microbiome plays a crucial role in host homeostasis, with implications for nutrition, immune development, metabolism, and protection against pathogens. Disturbance of the microbiome by microbial invasion can be negative or positive: invasions of opportunistic pathogens can cause disease while dysbiotic states need invasions to recover. However, the complexity of the microbiome challenges our understanding of what factors determine the ability of microbes to invade. In this study, we measure interactions between members of a synthetic community of prominent gut bacteria using supernatant assays, which quantify the growth of one species in the cell-free culture medium of another. We measure relative abundances of co-cultures of up to four species to validate a generalized Lotka-Volterra model parameterized with these supernatant assays. We predict differential invasion outcomes of the opportunistic pathogens *Escherichia coli* and *Bacteroides ovatus* based on their monoculture growth profiles and interactions with other species, and we experimentally confirm model predictions of invasion success. The predictive value of our model indicates that environmentally mediated interactions, e.g., through soluble chemicals, primarily determine co-culture abundances and invasion success. Furthermore, model analyses show that negative interactions within the resident community and neutral to positive interactions with the invading species promote invasion success, but the interactions toward the invading species dominate. Our validated approach opens the way for testing of interactions of human gut microbiome species, thereby developing interventions to avoid pathogenic overgrowth and therapies to enhance health-benefitting invasions.

**IMPORTANCE:**

The stability of the human gut microbiome is crucial for host health, with opportunistic pathogen invasions causing diseases and healthy strain replacements needed for recovery. The microbiota’s complexity complicates the understanding of invasion outcomes. This study uses a 10-species synthetic community of common gut microbiota to predict stable communities and invasion success. We grow cells in the growth medium of other species with the cells removed to parameterize a computational model, accurately predicting community composition up to four species and invasion success of *Escherichia coli* and *Bacteroides ovatus*. Our findings show that interactions through soluble compounds in the environment dictate co-culture growth and invasions. Furthermore, model analysis shows that interactions within the resident community and toward the invader are both important, but the latter dominate. These results pave the way for larger-scale studies to characterize gut microbiome interactions and properties that resist invasions, potentially benefiting health through improved probiotics and fecal microbiota transplants.

## INTRODUCTION

The human gut microbiota is important in host health, including resistance to invasion by opportunistic pathogens. A healthy microbiota is often characterized by high species diversity and temporal stability, while an unstable or disrupted microbiota is linked to a range of health issues, including inflammatory bowel disease and other gastrointestinal disorders (1–8). Overgrowth of opportunistic pathogens can lead to diseases, such as *Clostridioides difficile* infection, causing antibiotic-associated diarrhea and pseudomembranous colitis (9); *Candida* overgrowth, causing gastrointestinal candidiasis and potentially invasive candidiasis (10); small intestinal bacterial overgrowth, causing bloating, malabsorption, and nutrient deficiencies (11); expansion of Proteobacteria/Enterobacteriaceae, driving intestinal inflammation and increasing the risk of bacterial translocation, sepsis, or worsened inflammatory bowel disease (12); and *Fusobacterium nucleatum* overgrowth, which has been linked to colorectal cancer progression (13). Strategies to treat some of these diseases by fecal microbiota transplantation or targeted probiotic administration rely on engraftment, the successful integration of beneficial microbes into existing communities, which might be aided by previous administration of antibiotics (14). Since the efficacy of intervention such as fecal microbiota transplantation in inflammatory bowel disease is limited (15), it is crucial to understand how microbial species can successfully engraft in existing gut microbial communities.

Invasions in microbial communities have been studied with both theoretical and experimental approaches, aiming to understand which community properties affect invasion. Invasion can be classified as successful, with the resident community retaining its diversity (augmentation) or not (displacement), or as unsuccessful, with maintenance of the resident community (resistance) or not (disruption). In theoretical ecology, greater species richness and stronger competition have been shown to promote invasion resistance (16). More recently, ecological concepts have been applied to microbiology and human microbiota (17, 18). For example, increased diversity has been linked to invasion resistance in microbial communities (19). However, experimental support for the relationship is mixed, leading Hu et al. (20) to propose that it is the dynamic regime of the resident community—oscillations versus stable states—that better predicts invasion success. The outcome of microbial invasion is not solely determined by antagonistic interactions; facilitative interactions, where resident microbes assist invaders by altering environmental conditions or sharing metabolites, can promote successful invasions with either augmentation or displacement (21). Together, these studies highlight how important the specific ecological context is for invasion outcomes. Here, we investigate which properties affect invasion in a community of common human gut microbes using a combined experimental and computational approach.

Synthetic communities (SynComs) are useful tools to understand complex communities, capturing some of the complex interactions but retaining enough simplicity for experimental tractability (22, 23). SynComs of the human gut microbiota are used in many studies (24–31). We selected 10 abundant human gut microbes to cover a broad range of functions of the microbiome. These species, *Akkermansia muciniphila, Bacteroides ovatus, Bacteroides thetaiotaomicron, Bifidobacterium adolescentis, Blautia obeum, Lachnospiraceae* spp*., Lactobacillus johnsonii, Faecalibacterium prausnitzii, Roseburia intestinalis*, and *Escherichia coli*, spread over the phyla present in the western human gut and have been characterized in terms of their association with health or disease (32–34) (Fig. S1). They exhibit complementary metabolic capacities centered on the fermentation of polysaccharides and mucins, leading to the production of short-chain fatty acids (SCFAs), such as acetate, propionate, and butyrate (35, 36) and contain obligatory anaerobes (members of the *Bacteroides, Blautia, Lachnospiraceae, Faecalibacterium*, and *Bifidobacterium*) as well as facultative anaerobes capable of growth under microaerophilic conditions (*Lactobacillus johnsonii* and *Escherichia coli*) (37). Altered abundance or loss of these species has been linked to metabolic and inflammatory disorders including obesity, type diabetes, and inflammatory bowel disease (38, 39). Moreover, our selection includes two opportunistic pathogens: *E. coli* and *B. ovatus*. Both are common members of the healthy gut microbiota. *B. ovatus* has been suggested to promote health in some conditions (40), but also been identified as a significant contributor to clinical anaerobic infections and bacteremia (41). Moreover, *B. ovatus* abundance is higher in patients with intestinal bowel disease than healthy patients (42), and patients with ulcerative colitis show a disproportionate systemic immune response to *B. ovatus* (43), leading to the qualification as opportunistic pathogen alongside *E. coli*.

Microbial interactions are driven mainly by metabolic exchanges, the production of toxins, and direct physical contact. Supernatant experiments, where other species are cultured in growth medium from which cells have been removed, enable the study of environmentally mediated interactions and are readily scalable, as measurements do not require discriminating between species. Such experiments have been used to characterize species interactions (44) and been shown to capture a major component of the interaction structure within communities (45–47). This last observation is consistent with the finding that species dynamics can, for the most part, be captured by a kinetic model parameterized with time-series metabolite measurements (27). With only abundance measurements, species interaction models, such as Lotka-Volterra competition models, or its generalizations can be used to predict species dynamics. These models come with inherent limitations, such as limited predictability of batch cultures and the exclusion of higher-level interactions (48, 49). Despite these shortcomings, they have been used to predict dynamics in a human gut microbiota community and co-cultures of pathogens (28, 50). Moreover, they enable a test of the environmental mediation hypothesis: If models derived solely from supernatant assays accurately predict co-culture dynamics, it indicates that inter-species interactions are driven predominantly by diffusible chemical factors rather than physical or direct contact mechanisms.

Here, we use conditioned media assays, a form of supernatant assays, to describe the environmentally mediated species interaction structure of our SynCom. Next, we use a generalized Lotka-Volterra model to predict co-culture growth and validate these results with co-cultures of up to four species. Subsequently, we predict and validate species invasions in stable communities and elucidate the most important general properties of the invader and resident community that predict invasion success.

## RESULTS

### Negative interactions dominate between SynCom members

**Fig 1:**
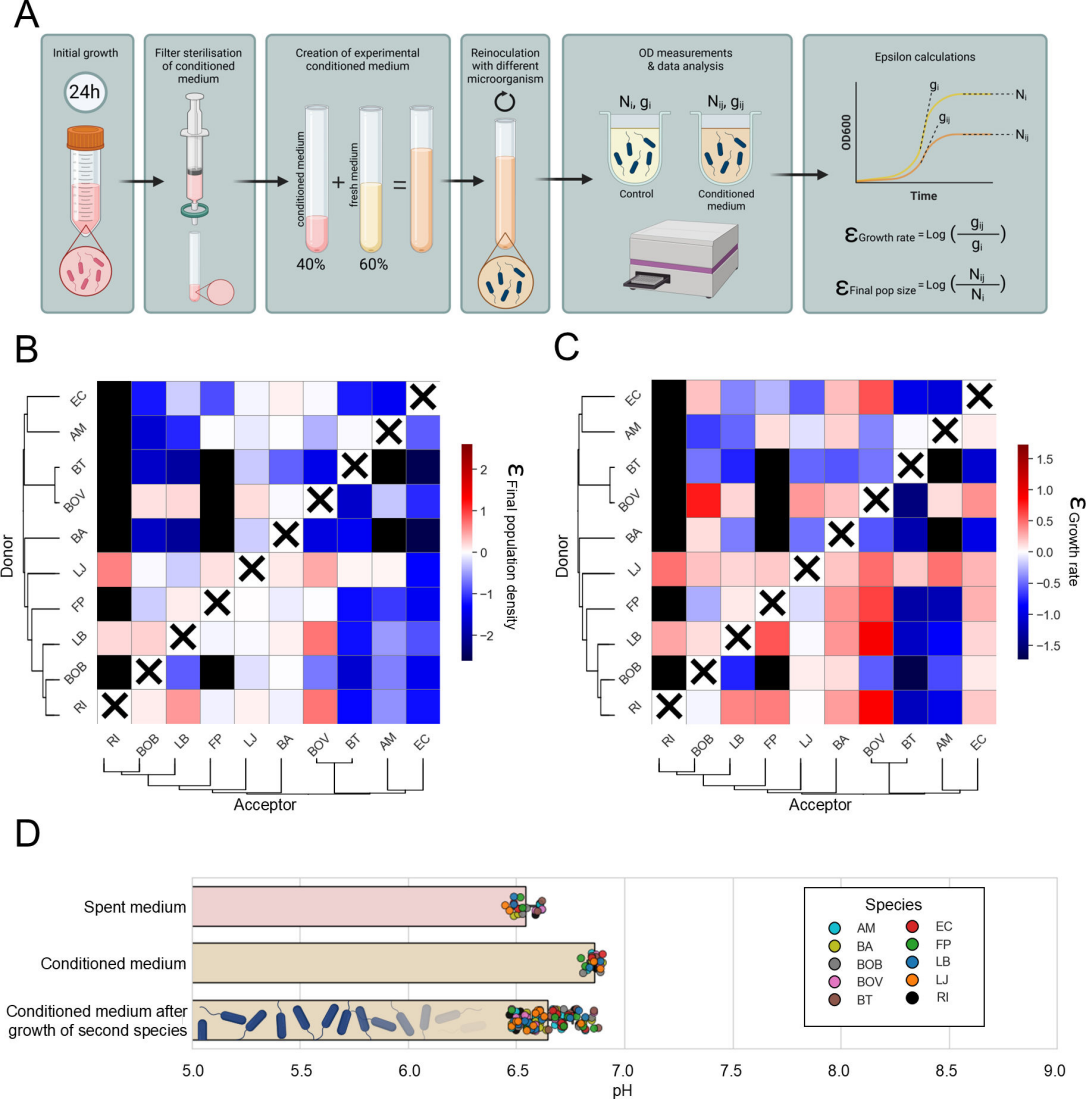
Interactions on growth rate and final population density measured by supernatant assays. (**A**) Experimental setup to test the invasions and calculation of the interactions. (**B**) Heatmap of the final population density interactions (the log of the final population density in conditioned divided by growth in YCFA medium). Species are ordered by phylogenetic distance. Black boxes denote that there was no growth of the organism in the conditioned media. Species are not assessed in their own conditioned media. (**C**) Heatmaps of the growth rate interactions (the log of the maximum growth rate in conditioned divided by growth in YCFA medium). (**D**) pH of the spent medium, the conditioned medium (60% spent medium and 40% YCFA) and the conditioned medium after growth of a second species. Individual dots are separate measurements, with the color indicating the last grown species.

To investigate the environmentally mediated interactions, we first created spent medium from all our SynCom members by growing them in YCFA medium until stationary phase and then filtering to remove the bacteria. We generated growth curves for each bacterial species in a conditioned medium composed of 40% spent media from other bacterial species and 60% fresh YCFA medium to assess species growth characteristics and interactions. We designated the bacterial species whose spent media was used as the “donor,” and the species that was inoculated in the conditioned medium as the “acceptor.” Overall, we see that negative interactions, which could be caused by e.g., resource competition or toxin production, dominate between the tested species (Fig. 1; Fig. S2A and B). Although the effects on growth rate and final population density correlate (Fig. S3), the effects of conditioned media on growth rate are less negative and more often positive than on maximum population density, suggesting that there are growth benefits of one species on another that do not affect the final population density. Conditioned media experiments cannot reveal the molecular mechanisms (44), but if the interactions are caused by symmetrical resource competition, we would expect an overrepresentation of reciprocal negative interactions, which we do not observe (Fig. S2C and D). Although known metabolic niches would predict clear donor–acceptor patterns, our results do not fully align with these expectations, suggesting that more than simple metabolite competition and exchange underlie the observed interactions. Specifically, we expected acetate-consuming species, such as *L. bacterium*, *R. intestinalis*, and *F. prausnitzii*, to thrive in the spent medium of acetate producers, including *A. muciniphila*, *B. adolescentis*, *B. obeum*, *B. thetaiotaomicron*, and *B. ovatus*; however, the outcomes were highly variable, indicating that additional factors, such as metabolic regulation, cross-inhibition, or signaling processes, may influence these community dynamics. We tested whether the observed growth patterns could be driven by pH-mediated effects. However, the conditioned medium showed negligible variation in pH, ensuring a nearly identical starting point for all species (Fig. 1D). Furthermore, even after the growth of a second species, the total pH range across all experimental conditions remained within half a pH unit, likely because of the phosphate buffer in our YCFA medium, showing that pH was not a factor driving the interactions.

Grouping the organisms by their effect on other species distinguishes *L. bacterium* and *L. johnsonii* as relatively facilitating species (i.e., having a less negative or positive effect as donors), even excluding their effect on *R. intestinalis* (Fig. S4). We wondered if the Gram type could explain species interactions. Our results show that Gram-negative bacteria experience very negative interactions as acceptors, and Gram-positive bacteria are much less affected by other species (Fig. S5). The phylogeny of the species also does not explain the patterns seen in the heatmaps (Fig. 1), even closely related species, such as *B. ovatus* and *B. thetaiotaomicron*, show different effects as both donor and acceptor species. Finally, we checked whether the monoculture growth characteristics of a species correlate with their effects as donor and acceptor species and found that species with a high monoculture final population density tend to have stronger negative interactions (Fig. S6). This could indicate a trade-off between monoculture growth and competitiveness with other species.

### *Roseburia intestinalis* is unable to grow in most of the conditioned media

Surprisingly, three bacteria were not able to grow in conditioned media, namely *A. muciniphila* (in *B. adolescentis* and *B. th*etaiotaomicron), *F. praustnitzii* (in the same plus *B. ovatus* and *B. obeum*), and *R. intestinalis* (in 7 out of 9 conditioned media) (Fig. 1). Given that *R. intestinalis* failed to grow in many of the conditioned media samples, we considered the possibility of a batch effect. Therefore, we repeated the experiment with *R. intestinalis* as acceptor and *A. muciniphila*, *B. adolescentis,* and *E. coli* as donors with the same negative result. To test whether the growth inhibition was caused by toxins that kill *R. intestinalis*, we performed a spot on lawn assay. The data do not confirm killing activity of the supernatant of *A. muciniphila*, *B. adolescentis,* and *E. coli* (data not shown). The hypothesis that growth inhibition, rather than lethal toxin production, accounts for the lack of growth is supported by our observation that *R. intestinalis* is able to grow in co-cultures when both species are inoculated at low initial densities (data not shown). Interestingly, *R. intestinalis* was positively affected by the two donors that supported growth (Fig. 1). Because of the difficulty in estimating interactions when we did not observe growth, we removed *R. intestinalis* from our further analyses.

### Generalized Lotka-Volterra model can predict growth in co-cultures

**Fig 2:**
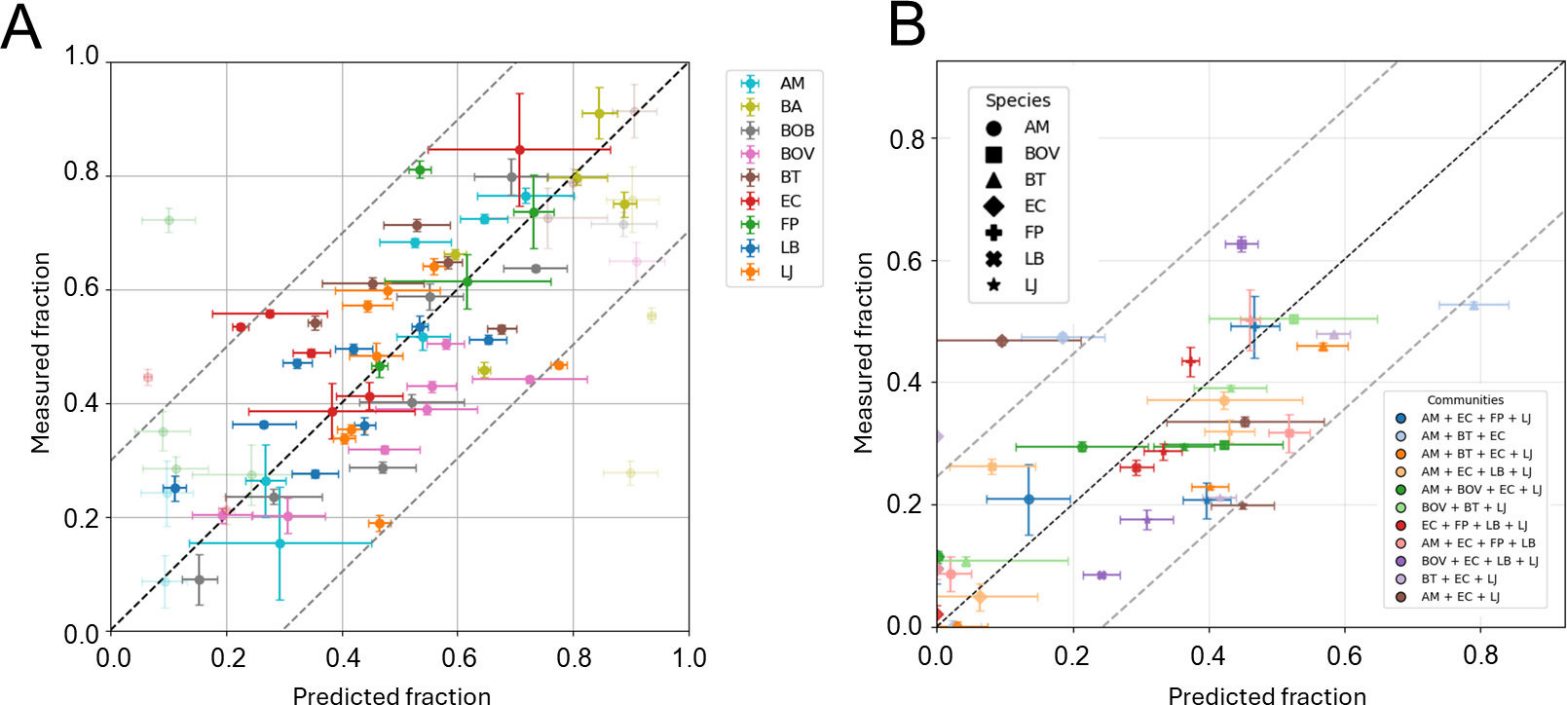
Validation of the model predictions with co-cultures of two to four bacteria. Predicted and measured species fractions are shown for co-cultures of two to four bacteria. Error bars represent standard deviations from 3 biological replicates (measured) and 100 sensitivity analysis samples (predicted). RMSE-based dotted bands indicate the 90% CI. (**A**) Bi-cultures (RMSE = 0.18, Pearson *r*² = 0.45). (**B**) Tri- and quad-cultures (RMSE = 0.14, Pearson *r*² = 0.50).

To test whether the growth in spent media reflects co-culture growth, we used a generalized Lotka-Volterra model to predict the equilibrium densities of co-cultures based on their interactions from the conditioned media (model adapted from de Vos et al. [45]). As the model equilibria depend solely on the final population density interactions, we refer to these as species interactions in the rest of our paper and will specify when we refer to the growth rate interactions. To validate the predictions, we grew the 36 combinations of two species and measured their final relative frequencies with qPCR. Overall, the relative frequencies of the two species are well predicted by the model (Fig. 2A) and clearly improve compared with predictions from monoculture growth only (Fig. S8A). Exceptions to the good predictions were mainly cases where one species was not able to grow in the conditioned medium of another, which makes it difficult to calculate their interactions (shown opaque in Fig. 2A).

We grew 11 co-cultures of three and four species for 24 h and compared the final fraction with model predictions (Fig. 2B; Fig. S7). The generalized Lotka-Volterra model with interaction terms predicts the relative abundances which cannot be predicted from monoculture growth data only (Fig. S8B). Next, we estimated the possible stable communities these species could form by calculating model equilibria with all species present. We found 21.4% of the combinations to be stable, with a maximum of six species, and stable communities to have a distribution of interaction parameters that is slightly shifted toward less negative and more neutral interactions (Fig. S9). Species that have less negative donor interactions on average are more likely to be found in stable communities (Fig. S10).

### Opportunistic pathogens *E. coli* and *B. ovatus* show different abilities to invade

**Fig 3:**
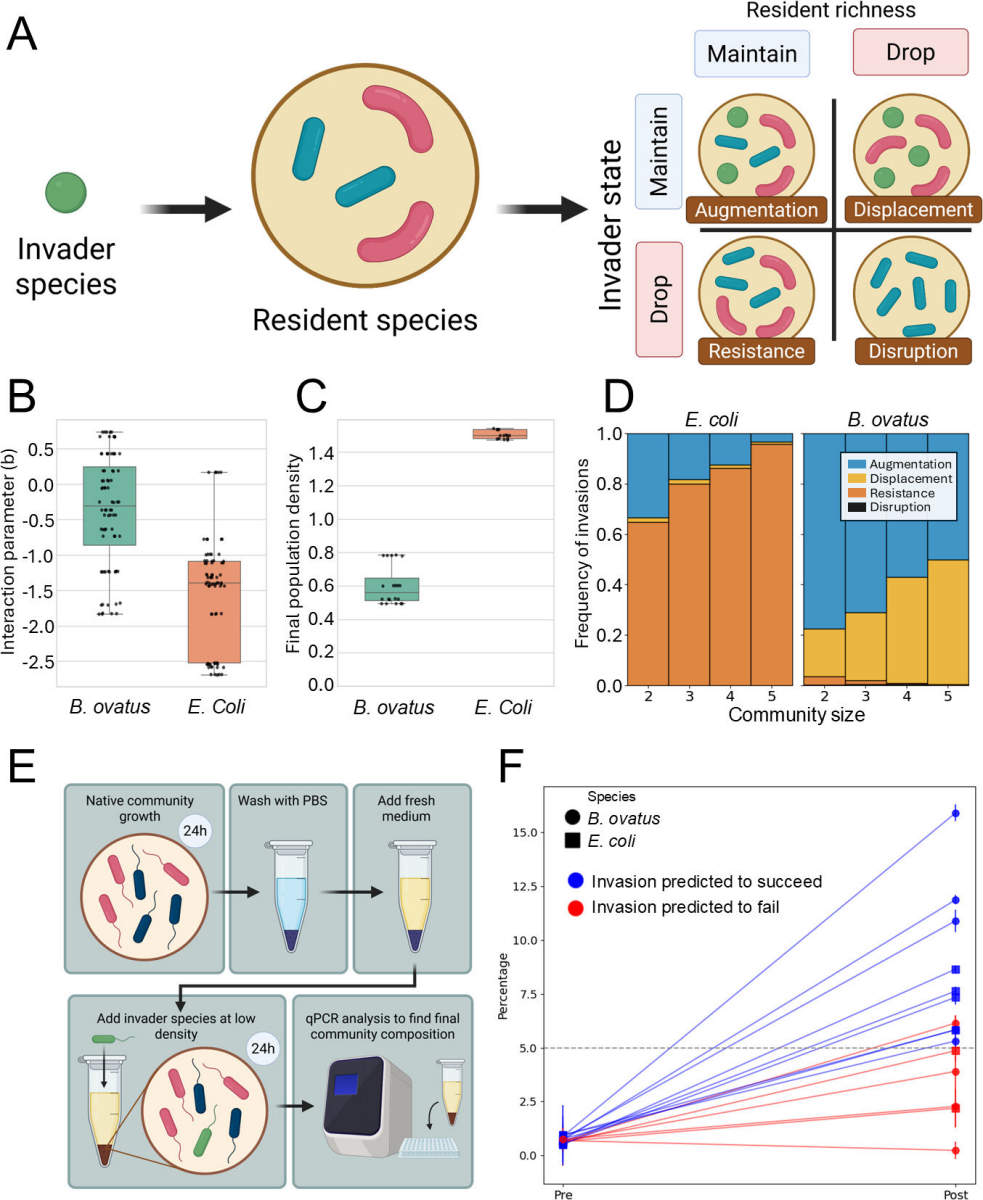
Invasion of *B. ovatus* and *E. coli* in stable communities. (**A**) Schematic of invasion types (adapted from reference 51). (**B**) Distribution of population density interaction parameter ($b_{ij}=\frac{N_{ij}}{N_{i}}-1$), with *B. ovatus* and *E. coli* as acceptor (i) and with all other species as donors (j) (nine replicas for each combination). (**C**) Final population density of *B. ovatus* and *E. coli* in monoculture (nine replicas). (**D**) Invasion outcomes of simulated invasions of *E. coli* and *B. ovatus* in predicted stable communities. (**E**) Schematic of the experimental procedure for testing the invasions. (**F**) Outcome of invasion experiments with *E. coli* (filled circles) and *B. ovatus* (filled squares) for predicted invasions (blue lines) and predicted resistant communities (red lines). The only predicted resistant community where the invader was just above the 5% threshold was repeated and then did show resistance (Fig. S11).

We next investigated whether these stable communities are susceptible to invasion, initially focusing on the opportunistic pathogens in our species set, *E. coli and B. ovatus. E. coli* has a high growth rate and final population density but is negatively affected by other species, while *B. ovatus* has a lower growth rate and final population density but is on average only slightly negatively affected by other species (Fig. 3B and C). We simulate invasions in stable communities based on the parameters derived from the conditioned media growth (Fig. 1). The two species show different invasion patterns. *E. coli* can only invade 24.1% of the stable communities in which it is not present, mostly resulting in augmentation (Fig. 3D). In contrast, *B. ovatus* can invade 97.6% of the stable communities resulting in augmentation (at low community sizes) or displacement (at high community sizes) (Fig. 3D). We tested the prediction of invasion success with invasion experiments, where the stable communities are concentrated and resuspended in YCFA medium plus a small amount of the invader (Fig. 3E). Relative growth of the invader was measured for 13 different communities. We predicted invasions using a 5% population threshold at 24 h and successfully predicted all observed invasions. Invaders in predicted-invasion communities grew significantly more than in communities where invasion was not predicted (Fig. 3F; Fig. S11).

### Invasion can be predicted from interactions within the community and between the community and the invader

**Fig 4:**
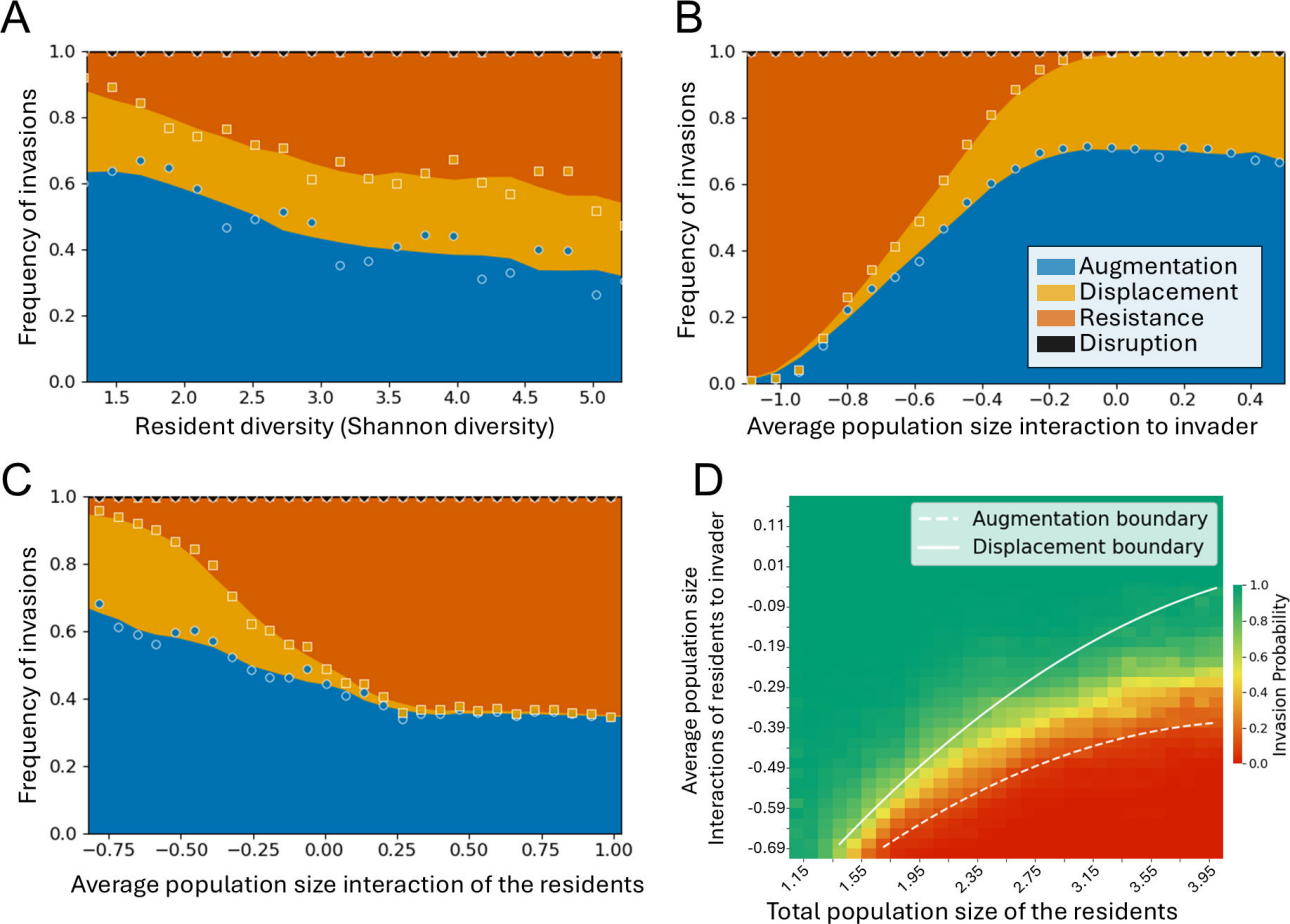
Community and invader properties affecting frequencies of different invasion types. (**A**) Increasing resident diversity (Shannon diversity index) decreases invasion success by decreasing the frequency of augmentation, while displacement frequency remains almost unaltered. (**B**) Negative interactions of the residents to the invader very strongly affect invasion success, reducing both augmentation and displacement. (**C**) More neutral and positive interactions between the resident species affect invasion success by limiting displacement and reducing augmentation. (**D**) Different invasion regimes are determined by the total population density of the residents and the average interactions of residents to invaders. The solid and dotted lines represent the boundaries of different invasion outcomes (displacements occur only above the solid line and augmentation only above the dotted line). In the region between the two lines, any successful invader only augments the resident community.

*B. ovatus* and *E. coli* exhibit distinct invasion patterns (Fig. 3D), reflecting their inherent differences in growth and interaction parameters (Fig. 3B and C ). These invasions were assessed in separate sets of stable communities, namely those that did not contain the invader. Moreover, we only assessed two species. To explore whether invader and community traits affect invasion success in an unbiased manner, we simulated species with uncorrelated characteristics (growth rates, maximum population densities, and interaction parameters) sampled from the observed distributions and tested their invasions in the full set of stable communities (see Material S3.2). Analyzing these invasions, we observe a shift from augmentation to resistance when the community diversity increases, while displacement remains mostly constant and disruption is almost absent (Fig. 4A). We scored several community and invader properties by their ability to predict invasions (Fig. S12A and B). The most predictive property for the invasion is the average interaction of the residents toward the invader, where negative interactions correlate with a resistant community, and neutral and positive values result in displacement and augmentation (Fig. 4B). The second most predictive property is the average interaction among the residents; with an increase in the interaction coefficient, displacement shifts to resistance, while augmentation is hardly affected (Fig. 4C). There is an interaction between the total population density and the interactions of the residents toward the invader that determines invasion success (Fig. 4D). At certain combinations of resident density and interaction strength, only augmentation is possible, meaning the invader can establish but does not replace a resident. Higher resident densities and stronger inhibitory interactions further reduce invader success, while lower densities or weaker interactions increase the likelihood of either displacement or integration. Other properties have very little impact on the invasion outcome or are strongly correlated with the aforementioned properties (Fig. S12). In conclusion, while both negative interactions among the residents and neutral to positive interactions toward the invader promote invasions, the interactions toward the invader dominate, explaining the often-reported conclusion that negative interactions decrease invasion success.

## DISCUSSION

We show that environmentally mediated pairwise interactions accurately predict co-culture growth and invasion success in a SynCom of prominent gut microbiota, indicating that such interactions are a key driver of co-culture dynamics and invasion success. Commonly used species-level traits, such as Gram classification and metabolic type, failed to predict interaction outcomes, emphasizing the need for context-specific interaction data from microbiomes. We further find that *E. coli* and *B. ovatus* interact differently with the other species, resulting in different invasion patterns. Invasion outcomes are determined primarily by growth interactions of resident species toward the invader and among residents, with only minor contributions from resident diversity and total population density, and an even smaller contribution from the invader’s effects on resident growth. The effect of the residents toward the invader dominates the invasion predictions, which is in line with previous research showing that negative interactions decrease invasion success (16, 20), experiments that show that the effect of the community on the invader predicts invasion outcomes (52) and that evolution of a community results in more resistance to invasion because the resident that evolves a more negative effect on the invader increases in density (53).

### Interactions cannot be predicted from putative metabolic interactions or Gram type only

Ideally, interactions between species can be predicted from physiological properties, such as metabolic phenotypes and Gram type. *E. coli, B. ovatus,* and *B. thetaiotaomicron* are capable of assisting fermenters via cross-feeding pathways (54, 55), but we only observe *B. ovatus* to have positive donor interactions (56). We also find that *B. ovatus* has three strong positive interactions as an acceptor species, for which we do not have a metabolic hypothesis. *F. prausnitzii, L. bacterium, B. obeum,* and *R. intestinalis* could be expected to be strong “acceptor” species since each can consume donor-excreted metabolites present in our community (acetate for *L. bacterium* and *F. prausnitzii*; lactate for *F. prausnitzii* and *R. intestinalis*; succinate for *B. obeum*), but we did not observe this in our conditioned media growth data (Fig. 1B and C). Neither can our interactions be explained from effects of the pH, as pH differences are very small, likely due to the buffer in our medium (Fig. 1D). Our results of the effect of Gram type on interactions contradict earlier findings by Zandbergen et al. (57), who found that Gram-positive donors tended to have stronger negative effects on other species’ growth compared with Gram-negative donors. This indicates that the effect of Gram type is highly dependent on the specific community or environment and cannot be used as a general predictor. Additionally, while one might expect the correlation we observed between the growth rate interactions and final population density interactions (Fig. S3), this was opposite to the observations from Zandbergen et al. (57). The data highlight the need for molecular physiological studies covering both primary and secondary metabolism.

### Strong response of *R. intestinalis* to other species

The inability of *R. intestinalis* to grow in conditioned medium seems to be a growth inhibitory rather than toxin-mediated interaction because we did not observe killing in a spot-on-lawn assay and did observe growth in co-cultures when both species start at low densities. Interestingly, such a sensitive species can thrive in a diverse community as the gut microbiome. The conditioned media it did grow in, donated by *L. johnsonii* and *L. bacterium*, had a positive effect on the growth characteristics of *R. intestinalis*. We hypothesize that this beneficial relationship is mediated by lactate and acetate production of *L. johnsonii* and *L. bacterium*, which are used as primary energy sources by *R. intestinalis*. Although both *L. johnsonii* and *L. bacterium* can produce folate and thiamine to which *R. intestinalis* is auxotrophic, these vitamins are present in the control medium making production of these compounds an unlikely explanation.

### Effects of community diversity and size and the effect on invasion resistance

Our model predicts co-cultures of up to four species (Fig. 2). Our predictions do not seem to need higher order terms, as was the case in previous studies (27, 58). A diverse gut microbiome is generally seen as healthy, which can be the result of enhanced invasion resistance. We do observe this relationship (Fig. 4A), and while the decrease in augmentation with community diversity agrees with the result of (21), we do not see the increase of displacement they report, perhaps because of the inhibitory interactions which we observe more in our community compared with their model.

### Individual versus environmental effects as predictors of invasion success

Previous studies have shown that more negative interactions lead to more invasion resistance (16, 20). While we observe similar results for more negative interactions of the resident species toward the invader, we find the opposite for negative interactions among the residents (Fig. 4B and C). These previous studies have only focused on negative interactions, and in our communities, we observe positive interactions, although limited. In a previous computational study with a kinetic model, the fraction of facilitative interactions (i.e., positive interactions) toward the invader was shown to increase invasion success (51). Extending the analysis from Hu et al. (20) to positive interactions, we observe a non-monotonic response of the invasion success to the average interactions, with maximal invasion at interactions closer to zero (Fig. S13A). Moreover, we notice that for fixed interactions with the invader, more negative interactions indeed lead to lower invasion success, similar to our findings (Fig. S13B). We can interpret our approach and theirs as studying idiosyncratic interactions versus environmental interactions: by decoupling the resident interactions with the interactions toward the invader, we study the properties of individual differences. Drawing those interactions from the same distributions (as in references 16, 20) can be interpreted as the effect of the environment on interactions. While many interactions can indeed be explained by environmental effects, it remains important to separate intra-resident interactions and resident-to-invader interactions, since interactions may also arise from species-specific mechanisms, such as toxin production, specialized cross-feeding, or other unique physiological traits.

### Limitations of the selection of species and culture conditions

The lack of correlation between known metabolism and measured interactions could result from the choice of medium, which does not directly reflect the gut environment and was selected to allow all species to grow and be measured in the same medium. The lack of complex carbon sources, such as mucin, limits the possible cross-feeding interactions. We only found stable communities of up to five species, which is small compared to the microbial diversity observed *in vivo*. This could be because the model assumes a homogeneous environment and neglects spatial heterogeneity, host factors, and differential objectives, such as adhesion versus growth. Indeed, spatial heterogeneity is a driver of gut microbial assembly with distinct microbial compositions and interaction networks across intestinal regions (26, 59), and host factors are known to influence colonization and community stability (60, 61). Moreover, we only analyze 10 species, and that could limit the community size by having limited interactions profiles. Testing the interactions between a wide range of species from a specific environment and in a range of environments would paint a more complete picture of the *in vivo* conditions and would allow for testing invasion in more diverse resident communities.

In conclusion, we showed that our methods can predict which species are likely to successfully engraft into existing synthetic communities. Future research can test whether these predictions can be extended to *in vivo* settings, potentially paving the way for personalized or targeted probiotics or fecal transplant donors. Being able to predict invasions that either enhance community resistance to pathogens or help restore disturbed microbiome states could substantially improve the success of such treatments.

## MATERIALS AND METHODS

### Bacteria and growth conditions

Bacterial strains were obtained from the DSMZ culture collection, and their identity was confirmed through 16S rRNA sequencing using universal primers, 16S forward (5′-AGAGTTTGATCCTGGCTCAG-3′); reverse (5′-ACGGCTACCTTGTTACGACTT-3′). In this study, the following bacterial strains were used: *Akkermansia muciniphila* (DSM 22959), *Bacteroides ovatus* (DSM 1896), *Bacteroides thetaiotaomicron* (DSM 2079), *Bifidobacterium adolescentis* (DSM 20083), *Blautia obeum* (DSM 25238), *Escherichia coli* (DSM 18039), *Faecalibacterium prausnitzii* (DSM 107838), *Lachnospiraceae bacterium* (DSM 24404), *Lactobacillus johnsonii* (DSM 10533), and *Roseburia intestinalis* (DSM 14610). The strains were then grown in YCFA media containing a phosphate buffer under anaerobic conditions using an anaerobic chamber filled with anoxic gas (10% H_2_, 80% N_2_, 10% CO_2_) set at 37°C, unless mentioned otherwise. The Sankey diagram for Fig. S1 was made using Sankeymatic (62).

### Conditioned medium preparation

We prepared conditioned medium according to De Vos et al. (39). Bacteria were inoculated in YCFA medium and grown for 24 h. After 24 h, we measured the optical density (OD) of the culture to ensure that the bacteria had surpassed log phase growth (OD > 1). Bacteria were filtered using a 0.2-µm filter to obtain the spent medium. We then prepared conditioned medium by mixing 60% fresh YCFA medium with 40% of spent medium.

### Growth of bacteria in conditioned medium

For the growth experiments, bacteria were pre-cultured for 16 h or until they reached logarithmic phase. The bacteria were then diluted to an OD of 0.05 in conditioned medium. All 10 bacteria were cultured in all other nine conditioned media, resulting in a total of 90 combinations. The growth of the bacteria was monitored using a Byonoy plate reader at a wavelength of 595 nm for 24 h in an anaerobic workstation (Whitley A35 Workstation, Don Whitley Scientific) under anaerobic conditions (10% H_2_, 10% CO_2_, 80% N_2_) at 37°C.

### Co-culture growth experiments

To grow the co-cultures, the bacteria were first individually pre-cultured for 16 h or until they reached logarithmic phase. Using OD-CFU correlations, the bacteria were combined using an equal number of CFUs and subsequently diluted to a final combined OD of 0.05. All co-cultures were grown for 24 h in 8 mL flasks. After 24 h, the OD was measured and the samples were prepared for downstream applications.

### Invasion assay

Initial consortia were cultivated for 24 h starting at an OD600 of 0.05. Simultaneously, a preculture of the invading species was grown under identical conditions. After 24 h, the OD600 of the initial consortia cultures was measured. The bacterial cultures were centrifuged at 13,000 × *g* for 5 min, and the supernatant was discarded. The cell pellets were resuspended in fresh YCFA medium. The invading species was washed twice with PBS, and its OD600 was measured. The washed invading species was then introduced into the resuspended initial consortia at a final OD600 of 0.05. Followed by an additional 24-h incubation period. Total DNA was isolated from the cultures and then used for the qPCR assay.

### DNA isolation

DNA isolation was performed using a modified phenol-chloroform method. Co-cultures were grown as described above and centrifuged at max speed to pellet the cells. The pellet was resuspended in elution buffer (10 mM Tris-HCl, pH 8.5). The suspension was then mixed with an equal volume of phenol:chloroform:isoamyl alcohol (25:24:1) to separate the DNA from other cell components. The mixture was centrifuged, and the DNA-containing aqueous layer was transferred to a new container. The DNA was subsequently precipitated by adding ethanol and ammonium acetate to a final concentration of 0.75 M. The DNA was collected by centrifugation, washed with 70% ethanol, and dissolved in elution buffer. The purified DNA was then quantified using spectrophotometry and stored at −20°C for downstream applications.

### qPCR analysis of community composition

Primers targeting the bacterial species of interest were designed using primer3plus (https://www.bioinformatics.nl/cgi-bin/primer3plus/primer3plus.cgi) to amplify either a specific region of the bacterial 16S rRNA gene or a specific gene for the focal species and yield a PCR product of approximately 200 base pairs with a delta of 50 base pairs (Table S1). The designed primers were tested against all the other bacterial species used in this study to verify their specificity and exclude any potential false positives. PCR amplification was performed and followed by gel electrophoresis to confirm the expected product size and absence of nonspecific amplification. The SYBR green method was employed for quantitative PCR (qPCR) analysis. The qPCR reactions were performed in a total volume of 25 μL, containing 12.5 μL of SYBR green master mix, 1 μL of each forward and reverse primer (10 μM), 2 μL of template DNA, and 8.5 μL of nuclease-free water. Negative controls (no template DNA) were included in each run to monitor potential contamination. qPCR amplification was carried out using the Applied Biosystems 7300 Real-Time PCR with the following thermal cycling conditions: an initial denaturation step at 95°C for 5 min, followed by 40 cycles of denaturation at 95°C for 30 s, annealing at 60°C for 30 s, and extension at 72°C for 30 s. The fluorescence signal was measured at the end of each extension step. The quantification cycle (Cq) values were recorded in triplicate for each sample, and averages were used. Standard curves were generated using known concentrations of bacterial DNA containing the target sequence. The Cq values of the samples were compared with the standard curve to determine the initial bacterial load in each sample. Positive controls with known concentrations of the bacterial species were included to assess the efficiency and sensitivity of the qPCR assay. See Material S1.3 for the calculation of the errors.

### Modeling and data analysis

All data analyses were done in RStudio using R version 4.3.1 and Python version 3.11.4. Growth parameters were extracted through the growth rates package (63). Final population density interaction parameters, shown in Fig. 1B, were calculated as in de Vos et al. (45) with the following formula:


$$\varepsilon_{final\;population\;density}\;=\;log\left(\frac{N_{ij}}{N_{i}}\right)$$

where $N_{ij}$ is the final population density of species i in conditioned medium from species j, and $N_{i}$ is the final density of species i in YCFA medium.

Growth rate interaction parameters (Fig. 1C) were calculated analogously using the following:


$$\varepsilon_{growh\;rate}\;\;=\;log\left(\frac{g_{ij}\;}{g_{i}}\right)$$

where $g_{ij}$ is the growth rate of species i in conditioned medium from species j, and $g_{i}$ is the growth rate of species i in YCFA medium.

Co-culture predictions were made using the generalized Lotka–Volterra (GLV) equations incorporating a logistic growth formulation as in de Vos et al. (45), with minor modifications (see Materials S1 and S2 for parameters details, modifications and motivation of the GLV formulations). The final formula is as follows:


$$\frac{dX_{i}}{dt}=\;X_{i}\;g_{i}\;\max\left(10^{-3},1\;+\;\sum_{j}a_{ij}X_{j}\right)\left(1\;-\;\frac{X_{i}}{\min\left(\max\left(10^{-20},\;1\;+\;\sum_{j}b_{ij}X_{j}\right),2\right)}\right)$$

with $X_{i}$ the focal species and the interaction terms summing over the other species j with the growth rate interaction parameters $a_{ij}$ and the final population size interaction parameter $b_{ij}$. Interaction parameters were obtained from monoculture experiments conducted in both YCFA media and conditioned media from other species, according to the following formulas:


$$\;a_{ij}=\frac{g_{ij}}{g_{i}}-1\;and\;b_{ij}=\;\frac{N_{ij}}{N_{i}}-1.$$

Co-culture predictions were calculated from the equilibrium points of the sets of differential equations (Material S2.2). Error bars on the predictions were calculated with simulations with parameters drawn from a normal distribution using the standard deviations calculated from the measurement error (Material S1.2). To evaluate the quality of the fit between the predicted and measured data, the root-mean-square error (RMSE) was computed as a measure of the average deviation between predicted and measured values. In addition, the strength of the linear relationship between measured and predicted values was quantified using the Pearson squared correlation coefficient (*r*²). The Pearson correlation coefficient was obtained using the linregress function from the scipy.stats Python library. For the invasion assays, the model was simulated for 48 h and a threshold equal to the inoculum value was used to determine species persistence (see Material S3.1).

## Data Availability

All data and code are available in the GitHub repository: https://github.com/WortelLab/SynCom.

## SUPPLEMENTAL MATERIAL

Supplemental Figures, Table, and Material msystems.00113-26-s0001.pdf Fig. S1–S13, Table S1, and Materials S1–S3.

ASM does not own the copyrights to Supplemental Material that may be linked to, or accessed through, an article. The authors have granted ASM a non-exclusive, world-wide license to publish the Supplemental Material files. Please contact the corresponding author directly for reuse.
